# HAI-1 is an independent predictor of lung cancer mortality and is required for M1 macrophage polarization

**DOI:** 10.1371/journal.pone.0252197

**Published:** 2021-06-29

**Authors:** Stanley Borowicz, Daniel R. Principe, Matthew J. Dorman, Austin J. McHenry, Gautam Sondarva, Sandeep Kumar, Vijayalakshmi Ananthanarayanan, Patricia E. Simms, Ashley Hess, Ajay Rana

**Affiliations:** 1 Division of Hematology/Oncology, Loyola University Medical Center, Maywood, Illinois, United States of America; 2 Department of Medicine, Division of Hematology/Oncology Edward Hines Jr. VA Hospital, Hines, Illinois, United States of America; 3 Medical Scientist Training Program, University of Illinois College of Medicine, Chicago, Illinois, United States of America; 4 Department of Surgery, University of Illinois at Chicago, Chicago, Illinois, United States of America; 5 Department of Biochemistry and Molecular Genetics, University of Illinois at Chicago, Chicago, Illinois, United States of America; 6 Department of Pathology, Loyola University Medical Center, Maywood, Illinois, United States of America; 7 Loyola University FACS Core Facility, Loyola University Medical Center, Maywood, Illinois, United States of America; 8 Jesse Brown VA Medical Center, Chicago, Illinois, United States of America; University of Alabama at Birmingham, UNITED STATES

## Abstract

Non-small cell lung cancer (NSCLC) is the leading cause of cancer-related death worldwide. Though immune checkpoint inhibitors (ICIs) have revolutionized lung cancer therapy in recent years, there are several factors limiting the therapeutic efficacy of ICI-based immunotherapy in lung cancer. Recent evidence suggests that one such mechanism is the phenotypic shift of tumor-infiltrating macrophages away from an anti-tumor M1 phenotype and towards an anti-inflammatory and tumor-permissive M2 phenotype. Though this phenomenon is well documented, the means through which the lung tumor microenvironment (TME) usurps macrophage function are poorly described. Hepatocyte growth factor (HGF) is a known driver of both lung cancer pathobiology as well as M2 polarization, and its signaling is antagonized by the tumor suppressor gene HAI-1 (*SPINT1*). Using a combination of genomic databases, primary NSCLC specimens, and *in vitro* models, we determined that patients with loss of HAI-1 have a particularly poor prognosis, hallmarked by increased HGF expression and an M2-dominant immune infiltrate. Similarly, conditioned media from HAI-1-deficient tumor cells led to a loss of M1 and increased M2 polarization *in vitro*, and patient NSCLC tissues with loss of HAI-1 showed a similar loss of M1 macrophages. Combined, these results suggest that loss of HAI-1 is a potential means through which tumors acquire an immunosuppressive, M2-dominated TME, potentially through impaired M1 macrophage polarization. Hence, HAI-1 status may be informative when stratifying patients that may benefit from therapies targeting the HGF pathway, particularly as an adjuvant to ICI-based immunotherapy.

## Introduction

Lung cancer is the leading cause of cancer-related mortality in the United States, with a 5-year survival rate of 19.4% [1]. While overall outcomes are improving due to advances in both detection and therapy, prognosis is particularly poor for patients with metastatic disease, where 5-year survival is a dismal 5.2% [1]. Therefore, there is an unmet clinical need to better understand the molecular mechanisms that underlie lung cancer progression, particularly regarding barriers to immune checkpoint inhibition. Recent evidence suggests that the contributions of tumor-infiltrating macrophages to lung cancer progression are highly varied, often serving both pro and anti-inflammatory roles in lung cancer. Classically, M1 polarized macrophages express markers including Arginase-1 and CCR7. Contrastingly, M2 polarized macrophages are often considered anti-inflammatory, and express markers such as CD206 [2]. While the overall contributions of these macrophage subsets are still unclear, emerging evidence appears to support M2 macrophages as an important and underappreciated barrier to the efficacy of immune checkpoint inhibitors [3]. For instance, increased M2 infiltration into tumor nests predicted poor survival in non-small cell lung cancer (NSCLC), and outcomes were particularly poor for M2-rich tumors co-expressing PD-L1 [3]. Similarly, depletion of M2 macrophages using a melittin-based pro-apoptotic peptide reduced tumor burden in xenografted lung cancer cells [4]. As such, there is increased interest in the means directing M2 polarization in NSCLC in order to improve clinical responses to immune checkpoint inhibition, though the mechanisms directing the balance between M1 and M2 macrophages in NSCLC are poorly characterized.

To this end, hepatocyte growth factor (HGF) is an established modifier of the lung cancer tumor microenvironment. HGF ligands comprise the fundamental pathway responsible for regulation of tissue repair after injury, and dysregulation of HGF signals have been implicated in both tumor growth and metastasis. HGF seemingly has a central role in the induction and maintenance of an M2 phenotype [5], though the means through which HGF is upregulated in the TME is not well established. In our previous work, our group identified hepatocyte growth factor activator inhibitor type-1 (HAI-1) as a cell-autonomous tumor suppressor that negatively regulates the HGF pathway [6]. HAI-1 functions by binding to and inhibiting hepatocyte growth factor activator (HGFA), a trans-membrane protease expressed in epithelial cells that cleaves and liberates HGF so that it can become bioavailable [6].

Here, we first evaluate the expression of HAI-1 in human lung cancer datasets as well as primary patient specimens, and determine that there is a unique subset of lung cancer patients with loss of HAI-1 and a particularly poor prognosis. We next determine that, clinically, HAI-1 is inversely associated with M2 macrophages in lung cancer, and that knockdown of HAI-1 in tumor cells leads to increased M2 polarization *in vitro*. Finally, using primary NSCLC specimens, we determined that tumors with loss of HAI-1 expression have a near complete absence of M1 polarized macrophages. Combined, these observations suggest that loss of HAI-1 is a potential means through which tumors acquire an immunosuppressive TME though impaired M1 macrophage polarization, and identify a potential subset of patients who may be amenable to therapies targeting the actions of HGF pathway inhibitors such as crizotinib, particularly as an adjuvant to immune checkpoint inhibitors.

## Methods

### Genomic database analysis

As described in our previous studies [7, 8], The provisional lung cancer TCGA patient dataset, RRID:SCR_003193 (N = 230) or OncoSG dataset (N = 181) were downloaded (https://tcga-data.nci.nih.gov/tcga/) and visualized using cBioportal for Cancer Genomics, RRID:SCR_014555 [9, 10]. Detailed information regarding the patients, sequencing analyses, and protocols can be found on the webpage listed above. Using this dataset, survival was assessed using the Kaplan Meier method. Subsequent genetic analyses were restricted to only fully sequenced tumors and, per cBioportal, the mRNA values for each gene determined by comparing microarray data to the gene’s expression distribution in a reference population. All mRNA expression values are plotted in log scale unless other wise noted, and are displayed with the associated P and Spearmen (S) coefficient values.

### Histology, immunohistochemistry, and immunofluorescence

Lung cancer and adjacent normal tissue microarrays were purchased (Biomax, Derwood, MD, Cat #BC04119b, US Biomax, RRID:SCR_004295) and subjected to pathologic examination. Tissues were stained with hematoxylin and eosin (H&E) (Sigma Aldrich), or via immunohistochemistry (IHC). For immunohistochemistry, slides were deparaffinized by xylenes and rehydrated by ethanol gradient, then heated in a pressure cooker using DAKO retrieval buffer (DAKO, Santa Clara, CA). Endogenous peroxidases were quenched in 3% hydrogen peroxide in methanol for 30 minutes. Tissues were blocked with 0.5% BSA in PBS for 30 minutes and incubated with primary antibodies against HAI-1 (abcam, ab189511) or CCR7 (abcam, ab227768) at 1:50–1:200 overnight at 4°C. Slides were developed using HRP conjugated secondary antibodies followed by DAB substrate/buffer (DAKO). All human tissues were from commercially available cell lines and tumor microarrays and not subject to local IRB approval.

### Tissue slide counts, scores

All counts, measurements, and scores were performed on a commercially available patient tumor microarray (Biomax, Derwood, MD, Cat #BC04119b, US Biomax, RRID:SCR_004295) by a minimum of two blinded investigators. Slides were scored as described previously [11–14], with each investigator assessed the entire tissue core, and assigned a score of 0 (no expression), 1+ (uniform dim expression or focal areas of moderate expression), 2+ (uniform moderate expression or focal areas of strong expression), or 3+ (uniform strong expression). As described, all counts/measurements/scores were averaged and rounded to the nearest whole number.

### Macrophage induction and culture

THP-1 cells (ATCC TIB-202, RRID:CVCL_0006) were induced with 0.32 uM PMA for 24 hours. These induced M0 macrophages were then washed once with PBS and plated onto 10cm cell culture plates in RPMI supplemented with 10% FBS for 24 hours. Attached cells were then washed with PBS and then cultured in the presence of conditioned medium for approximately 40 hours, followed by 48 hours of standard culture medium (RPMI with 10% FBS) to allow for cell recovery prior to flow cytometry. Conditioned medium was generated using stably transfected H358 human lung adenocarcinoma cells (NCI-H358, RRID:CVCL_1559) with shRNA against HAI-1 or mock shRNA. H358 shHAI-1 or H358 shMock cells were cultured in serum-free RPMI for 24h after which this conditioned medium was spun down to remove any floating cells and then added to macrophage cell culture plates. Effective induction of M0 macrophages from THP-1 cells was confirmed via flow cytometry assessing CD68 expression.

### Flow cytometry

Cell surface immunofluorescence was performed as follows. The cells were incubated for 30 min on ice with saturating amounts of the antibody and were washed three times with Hanks balanced salts solution (without phenol red) containing 5% newborn calf serum. The cells were analyzed with a Becton Dickinson FACSCanto II instrument (BD Biosciences, San Jose, CA, USA, RRID:SCR_018056). Compensation was performed using fluorochrome-stained beads (eBiosciences, Waltham, MA) and calculated using FACSDiva software (BD Biosciences, RRID:SCR_001456). Data was analyzed using FlowJo X software (Tree Star, Ashland, OR). Immunostaining of induced THP-1 cells to confirm macrophage phenotype was performed with anti-CD68-PE-Cy7 antibody (Biolegend, RRID:AB_2562935). Immunostaining to assess macrophage phenotype was performed by incubating cells with anti-CCR7-PE antibody (BD Biolegend, RRID:AB_10916391) for M1 macrophages and anti-CD206-Alexa Fluor 488 (Biolegend, RRID:AB_571874) for M2 macrophages.

### Statistical analysis

Data were analyzed by either Student’s T-test, simple linear regression analysis, or ANOVA fit to a general linear model in Minitab express, the validity of which was tested by adherence to the normality assumption and the fitted plot of the residuals. Results were arranged by the Tukey method, and considered significant at p < 0.05 unless otherwise noted.

## Results

### HAI-1 is downregulated in NSCLC and is associated with poor clinical outcomes

**Fig 1:**
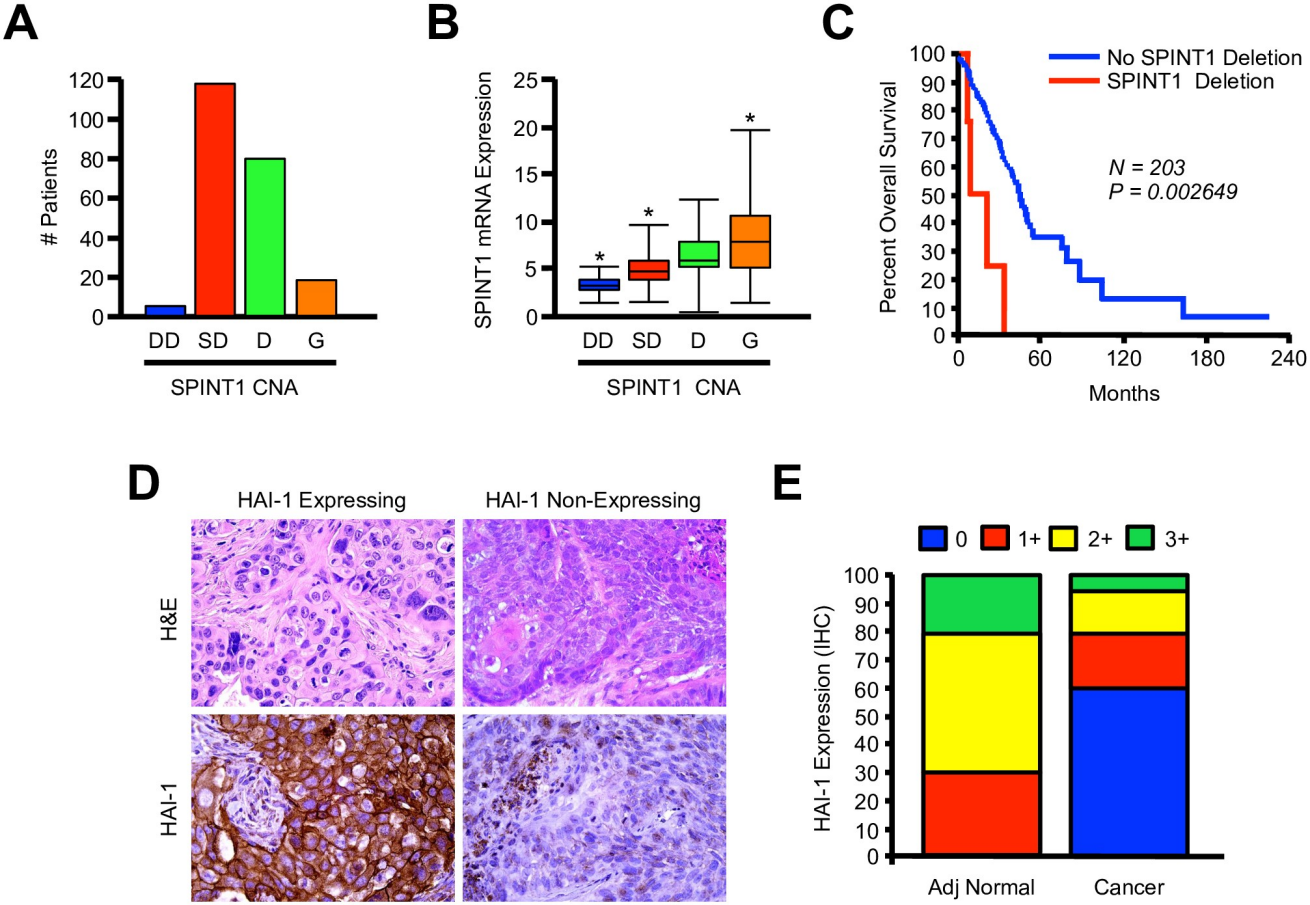
HAI-1 is downregulated in NSCLC and is associated with poor clinical outcomes. **(A)** Patient genomic profiles from the TCGA dataset were evaluated for copy number alteration (CNA) to HAI-1 (*SPINT1*), and arranged as either having deep deletion (DD), shallow deletion (SD), diploid (D), or gain of copy number (G). **(B)** Relationship between CNA and mRNA expression of *SPINT1*. **(C)** Kaplan-Meier curve showing overall survival for *SPINT1*-intact patients compared to those with deep deletion of *SPINT1*. **(D)** NSCLC patient tissues were stained either with H&E or by immunohistochemistry for HAI-1. Representative images are shown for HAI-1 expressing and non-expressing tissue sections. **(E)** HAI-1 expression was quantified as described, and compared between NSCLC and adjacent non-malignant tissues.

To determine a potential role for HAI-1 in NSCLC, we first evaluated the TCGA genomic database of NSCLC patients (N = 230) for both copy number and mRNA expression of the HAI-1 gene *SPINT1*. When evaluating copy number alterations (CNAs), of the 223 patients for which this information was available, we found that 3% of NSCLC patients showed deep deletion of *SPINT1*, and 53% had shallow deletion (Fig 1A). 8% of patients had a gain of *SPINT1* copy number, with the remaining 36% diploid for *SPINT1* (Fig 1A). This corresponded to changes in *SPINT1* mRNA expression, which was highest in patients with gain of copy number, and lowest in patients with deep deletion (Fig 1B). Interestingly, for the 221 patients for which survival data was available, those with genetic deletion had a particularly poor prognosis, with a median survival of 20.63 months compared to those with intact *SPINT1*, who had a median survival of 49.24 months (Fig 1C). Given these observations, we next evaluated the expression of the HAI-1 protein in 20 human NSCLC specimens, as well as 10 adjacent non-malignant tissues by immunohistochemistry. Consistent with the TCGA data, while all adjacent normal tissues were positive for HAI-1, 60% of NSLC specimens had loss of HAI-1 by IHC (Fig 1D and 1E).

### HAI-1 mRNA expression inversely associates with HGF and predicts for poor tumor immunogenicity

**Fig 2:**
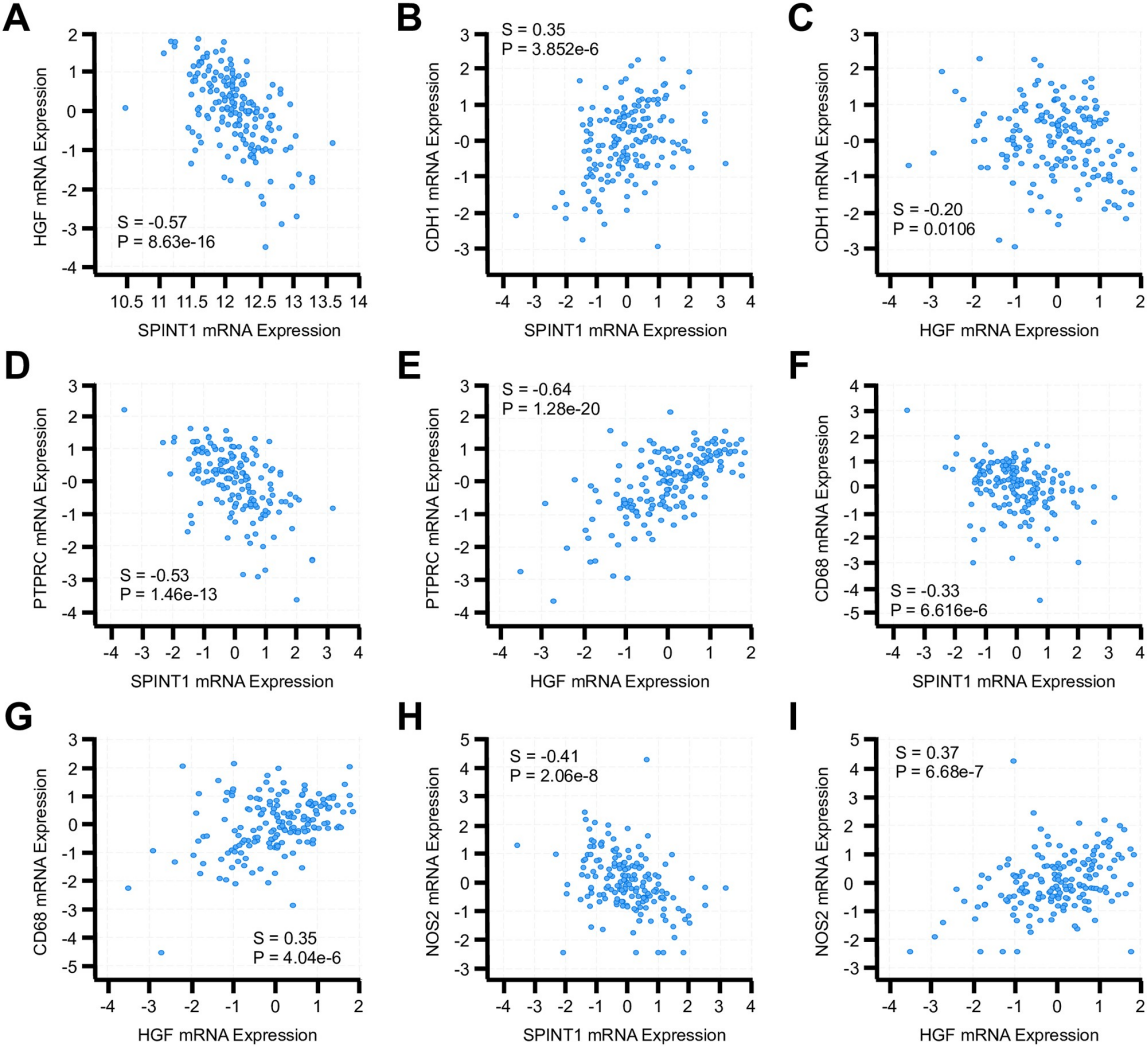
HAI-1 mRNA expression inversely associates with HGF and predicts for poor tumor immunogenicity. Patient mRNA profiles from the OncoSG dataset were evaluated for expression of HAI-1 (*SPINT1*) and *HGF*. The expression of these genes were related to **(A)** each other, **(B, C)** the epithelial surrogate *CDH1*, **(D, E)** the pan-leukocyte marker CD45 (*PRTPC*), **(F, G)** the macrophage marker CD68, or **(H, I)** the M2 macrophage marker iNOS (*NOS2*). The Spearman’s Correlation Coefficient (D) and P value of all potential interactions are displayed above.

Given the established role for HAI-1 as an inhibitor of HGF [6], we next evaluated the relative expression of *SPINT1* and *HGF* transcripts in the OncoSG genomic database of NSCLC patients (N = 181), as well as the mRNA expression of functionally related genes. Consistent with the role of *SPINT1* as an inhibitor of HGF signaling, we observed a highly significant inverse association between *SPINT1* and *HGF* mRNA (Fig 2A). We also observed a strong, positive association between *SPINT1* and the epithelial surrogate marker *CDH1* (Fig 2B), consistent with our previous observations in which HAI-1 staining localized predominantly to the neoplastic epithelium. Conversely, *HGF* mRNA expression was negatively associated with that of *CDH1*, and rather correlated with the expression of the pan-leukocyte antigen *PTPRC*, also known as CD45 (Fig 2C–2E). As HGF is an established driver of tissue repair and inflammation, we next evaluated the relationship between SPINT1, HGF, and the macrophage surrogate CD68. *SPINT1* mRNA expression was inversely associated with that of *CD68* (Fig 2C, P= 1.19x10^-8^), whereas *HGF* was also positively associated with *CD68* mRNA (Fig 2F and 2G). Though *HGF* strongly associated with *CD68*, *HGF* had only a weak association with the M1 polarization marker *CCR7* (S1A Fig), though *HGF* expression was more closely correlated to expression of the M2 polarization marker *NOS2*. Accordingly, *HGF* also was significantly co-expressed with a variety of M2-derived cytokines, including *TGFB2* and *IL10* (S1B and S1C Fig).

### Epithelial loss of HAI-1 leads to impaired M1 macrophage polarization *in vitro*

Given the apparent association between loss of HAI-1 and an M1-poor immune infiltrate, we next evaluated the contributions of epithelial HAI-1 loss to macrophage polarization *in vitro*. We first stably transfected H358 human lung adenocarcinoma cells with either a scramble shRNA control vector (shCtrl), or shRNA against HAI-1 (shHAI-1). After antibiotic selection, the reduction in HAI-1 was verified by western blot, we conditioned serum free media for 24 hours, supplemented media with 10% FBS, and transferred this media to non-polarized (M0), THP-1-induced macrophages.

**Fig 3:**
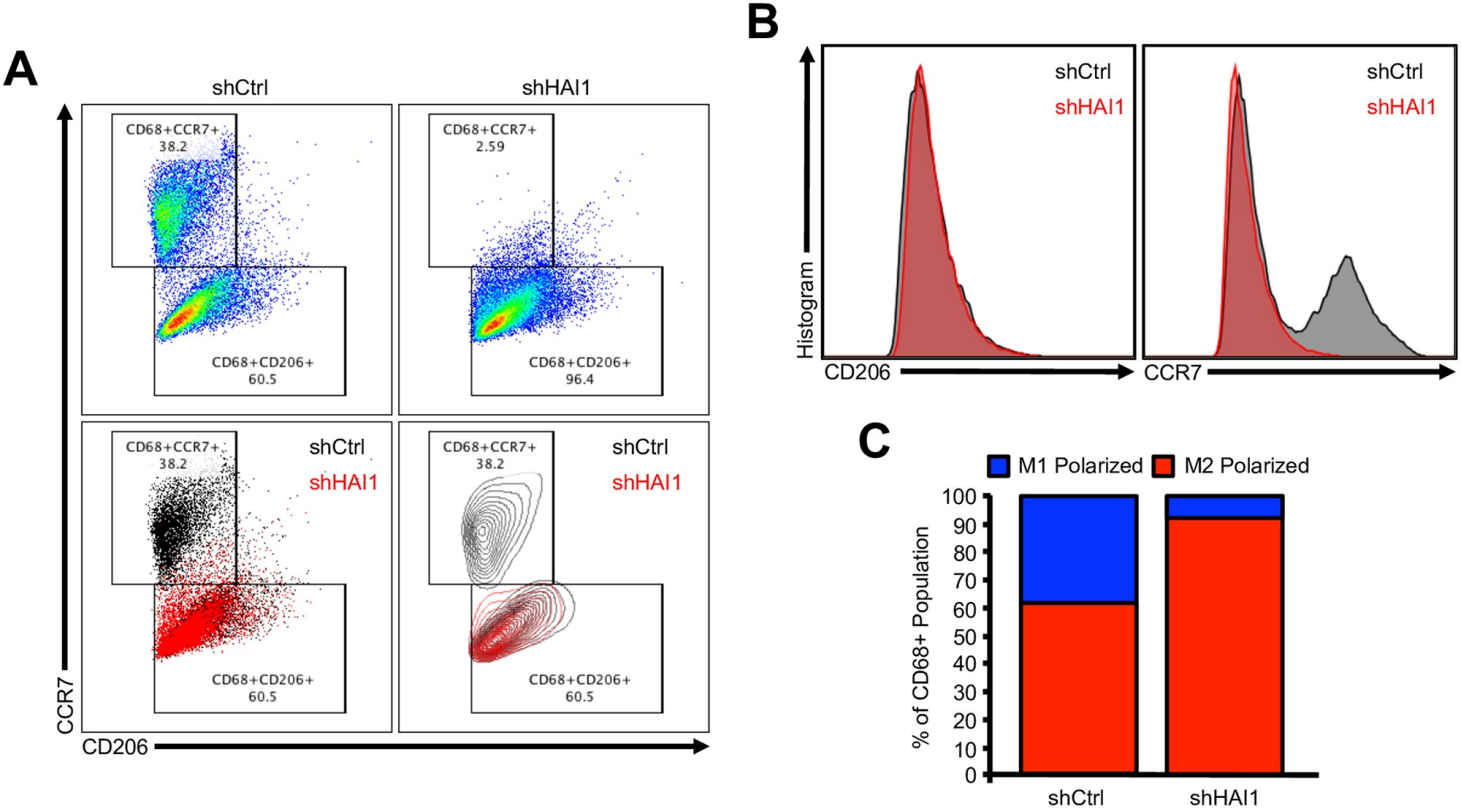
Epithelial loss of HAI-1 leads to impaired M1 macrophage polarization *in vitro*. **(A)** H358 human lung adenocarcinoma were transfected with either a scramble shRNA control vector (shCtrl), or shRNA against HAI-1 (shHAI-1). Conditioned media was collected and transferred to non-polarized (M0), THP-1-induced macrophages. After 48 hours, THP-1-induced macrophages were allowed to recover in control media for another 48 hours and analyzed by flow cytometry for the macrophage surrogate CD68, the M1 polarization marker CCR7, and the M2 polarization marker CD207. **(B)** Histogram plot showing the relative expression of CD206 or CCR7 in THP-1-induced macrophages cultured media from either shCtrl or shHAI-1 H358 cells. **(C)** Relative fraction of THP-1-induced macrophages displaying an M1 or M2 polarized phenotype following incubation in conditioned media from wither shCtrl or shHAI-1 H358 cells.

These M0 macrophages were grown in these respective conditioned medias for 48 hours, after which they were allowed to recover in control media for another 48 hours. At this point, cells were then collected and analyzed by flow cytometry for the macrophage surrogate CD68, the M1 polarization marker CCR7, and the M2 polarization marker CD207. Interestingly, both M1 and M2 populations were represented in M0 cells treated with shCtrl-conditioned media. However, M0 macrophages treated with shHAI-1-conditioned media had little to no M1 macrophages, with nearly all cells expressing CD207 (Fig 3A–3C).

### Loss of HAI-1 is associated with reduced M1-macrophage infiltration in microarray lung cancer specimens

**Fig 4:**
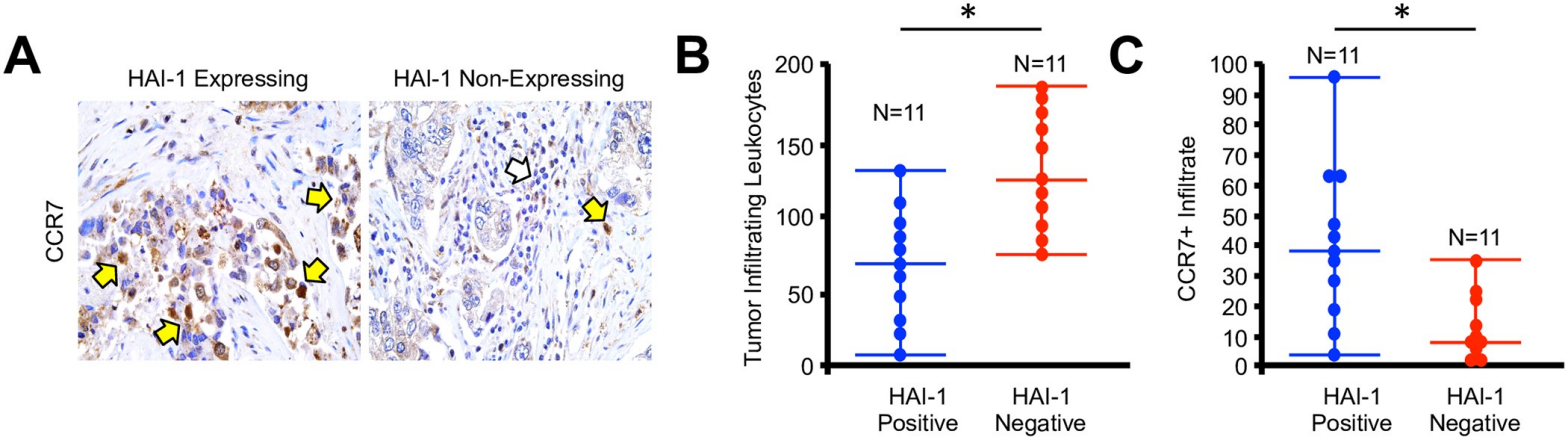
Loss of HAI-1 is associated with reduced M1-macrophage infiltration in microarray lung cancer specimens. **(A)** NSCLC specimens were stained for expression of the M1 macrophage surrogate CCR7, and representative images shown for HAI-1 expressing and HAI-1 non-expressing tissues. **(B)** The number of tissue infiltrating leukocytes per high power field was quantified and arranged by HAI-1 status. **(C)** CCR7 positive macrophages were quantified per high power field and arranged by HAI-1 status. (*p <0.05).

Given the apparent necessity of HAI-1 for M1 polarization, we next evaluated 20 human lung adenocarcinoma samples for expression of HAI-1 or CCR7 by immunohistochemistry. Consistent with our previous data, HAI-1 was lost in roughly 50% of patients. However, the majority with intact expression of HAI-1 had a robust CCR7+ immune infiltrate, whereas nearly all patients with loss of HAI-1 had little to no CCR7+ immune cells despite a modest increase in overall leukocyte infiltration (Fig 4A–4C).

## Discussion

**Fig 5:**
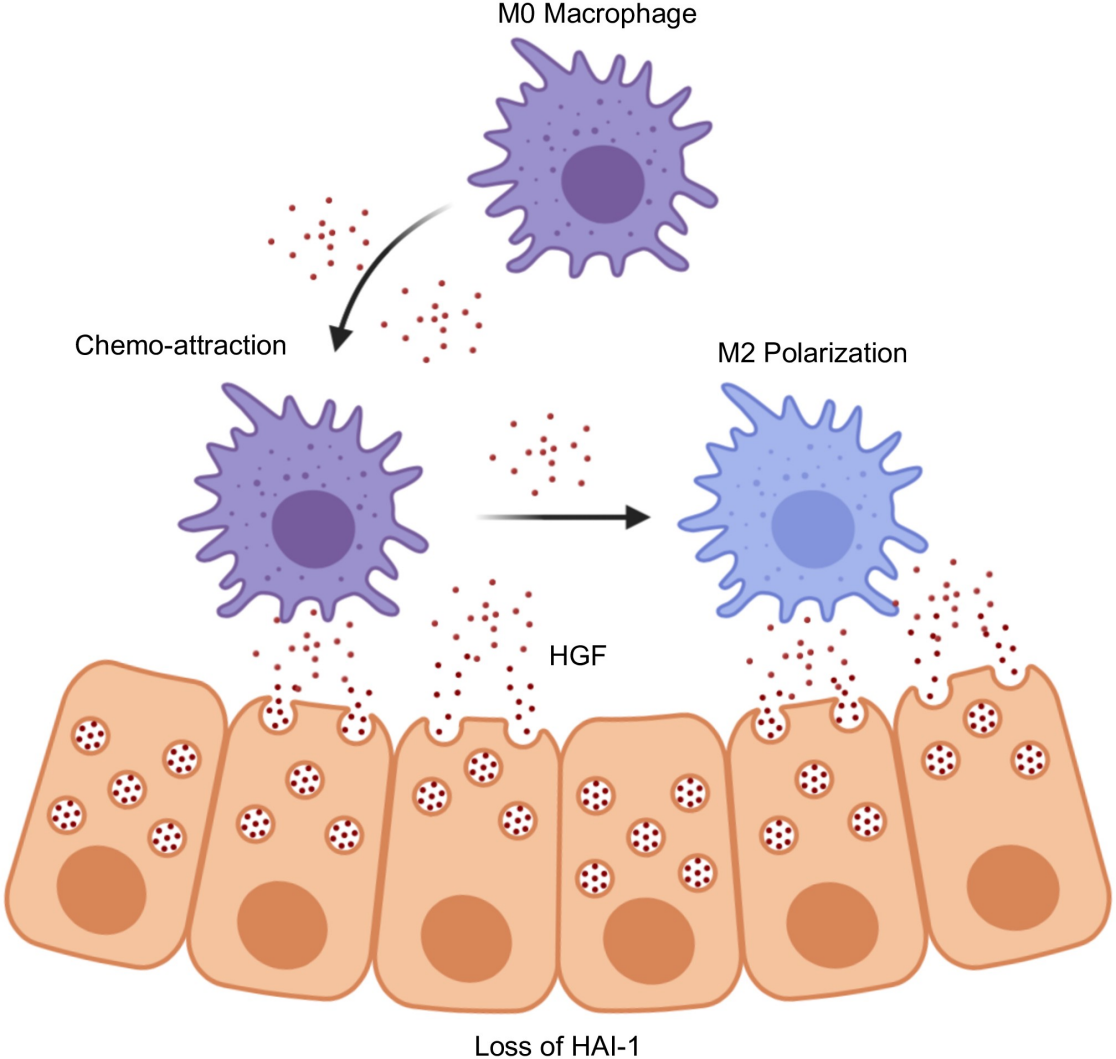
Schema describing the presumptive mechanism through which HAI-1 loss reshapes paracrine signals between lung tumor cells and infiltrating macrophages. In the proposed mechanism, genetic loss of HAI-1 (*SPINT1*) leads to the de-repression of HGF. The resultant increase in HGF leads to enhanced macrophage chemo-attraction. However, HGF as HGF is central to M2 polarization, this HGF-rich environment causes these tumor-infiltrating macrophages to favor a suppressive M2-polarized phenotype, thereby limiting their anti-tumor immune responses.

Here, we demonstrate that not only does loss of HAI-1 predict for poor clinical outcomes in lung cancer, but that HAI-1 also appears to have a pivotal role in directing the polarization of the tumor infiltrating macrophages (TAMs). Specifically, we found that lung cancers deficient in HAI-1 have a loss of CCR7-expressing M1 macrophages. Further, loss of HAI-1 in cultured tumor cells corresponded to an inability to induce an M1 phenotype in unpolarized macrophages (summarized in Fig 5). This is noteworthy, as the subversion of local immune responses is an essential step in lung cancer pathobiology [15]. Importantly, tumor cells develop myriad ways to escape T-cell-mediated cytotoxicity including the expression of negative immune checkpoints e.g. PD-L1, overexpression of soluble immunosuppressants, diminished antigen presentation, and a shift toward an M2-dominant macrophage infiltrate [16, 17].

As discussed, HGF has a central role in maintaining the M2 phenotype [5]. Here, we demonstrate that loss of HAI-1 leads to the increased expression of HGF mRNA in lung cancer samples, which likely links loss of HAI-1 expression to the observed loss in M1 polarized TAMs. The relationship between HAI-1 and HGF signaling is well established, particularly in tissue repair and wound healing. Classically, circulating pro-HGF is produced mainly by tissue fibroblasts, where it is biologically inactive until cleaved and activated by a variety of proteases such as hepatocyte growth factor activator (HGFA), which are also expressed by tumor cells [18]. As discussed, HAI-1 functions by binding to and inhibiting HGFA, thereby reducing the bioavailability of HGF [18, 19]. In the setting of HAI-1 deficiency, activated HGF exerts its effects on target cells via the MET receptor [20, 21]. Accordingly, MET receptor activation has been associated with poor prognosis in NSCLC [22], and copy number amplification of MET predicts for response to the tyrosine kinase inhibitor crizotinib [23]. However, effective targeting of the MET receptor alone has had limited clinical success in clinical trial [24].

Though HAI-1 loss and increased HGF/MET signaling has been implicated in a variety of cell processes such as proliferation [25] and epithelial to mesenchymal transition [26], our data also suggests that enhanced HGF signaling secondary to HAI-1 loss may have important effects in the evasion of immune surveillance. TAMs are a newly emerging barrier to the efficacy of immune checkpoint inhibitors. For instance, response to PD-1 inhibition relies requires PD-L1 expression on tumor cells, as well as PD-1 expression on TAMs [27]. The role for HAI-1/HGF signaling in these events is now becoming clear. Loss of HAI-1 and the resultant increase in MET signaling leads to increased PD-L1 expression in renal cell carcinoma [28]. Similarly, the MET inhibitor crizotinib leads to the downregulation of PD-L1 in MET-amplified NSCLC cells [29].

In light of these observations, HAI-1 loss warrants further exploration as a potential biomarker for MET inhibitors such as crizotinib, particularly in combination with immune checkpoint inhibitors. This approach is currently under early investigation, as Phase I trials are now evaluating crizotinib and the PD-1 inhibitor pembrolizumab in ALK, ROS1, and MET-driven NSCLC [30, 31]. However, through further explorations of HAI-1/HGF signaling and its roles in reshaping local immune responses, it may be possible to substantiate HAI-1 loss as a biomarker for such an approach, thereby identifying additional NSCLC patients who would derive clinical benefit from such combination strategies.

## Supporting information

S1 Fig HGF expression associates with an M2 macrophage gene signature. Patient mRNA profiles from the OncoSG dataset were evaluated for expression of HGF, which was related to **(A)** the M1 macrophage marker CCR7 as well as the M2-associated cytokines **(B)** TGFB2 and **(C)** IL10. The Spearman’s Correlation Coefficient (D) and P value of all potential interactions are displayed above.
(TIF)

## Abbreviations

HGF: Hepatocyte growth factor
HGFA: Hepatocyte growth factor activator
NSCLC: Non-small cell lung cancer
TAMs: tumor infiltrating macrophages
TME: Tumor microenvironment
