# Supplementary figures and images for: HAI-1 is an independent predictor of lung cancer mortality and is required for M1 macrophage polarization
Source: PLoS One. 2021 Jun 29;16(6):e0252197. doi: 10.1371/journal.pone.0252197 (PMC8241049; doi:10.1371/journal.pone.0252197)

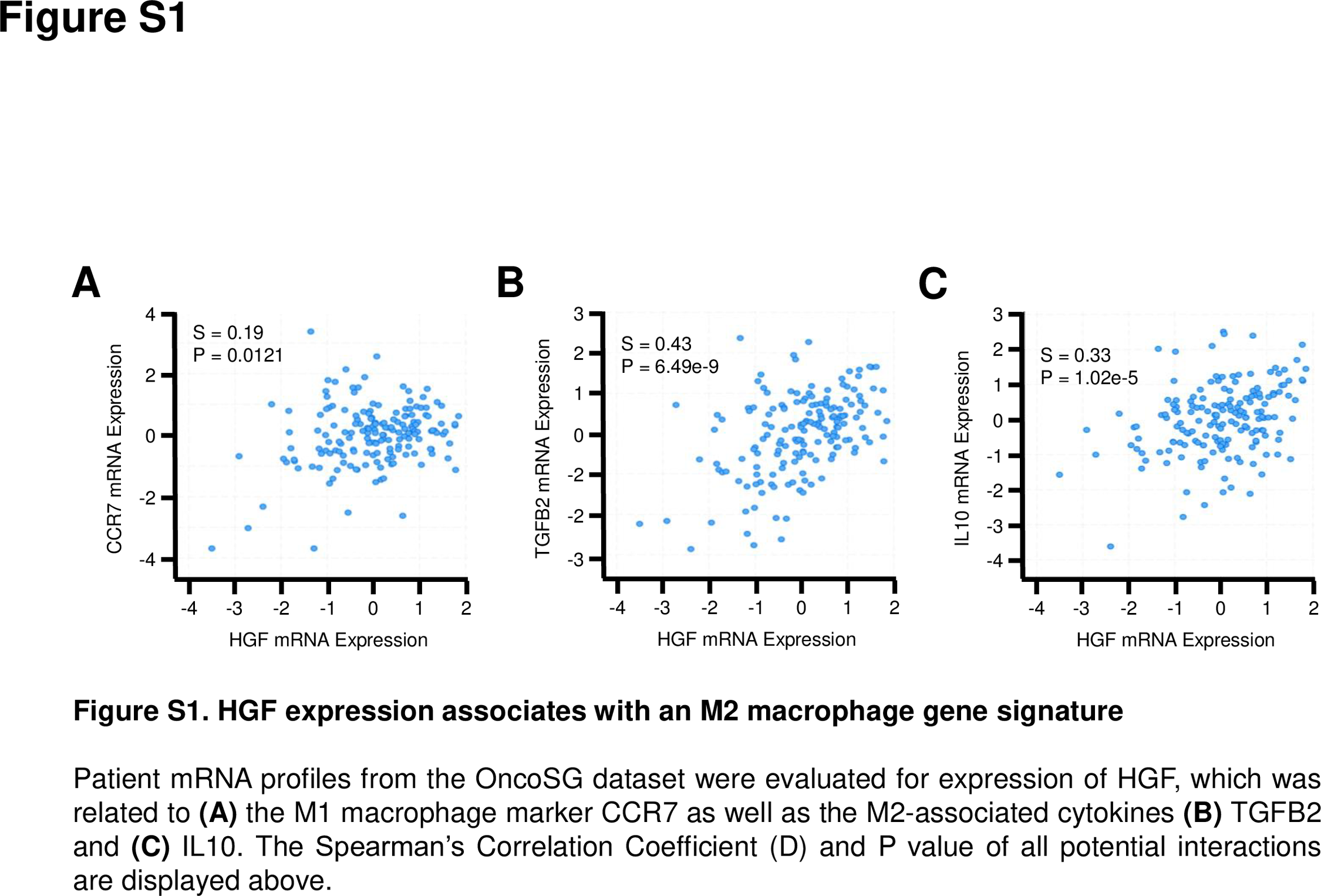

Supplement: S1 Fig — Patient mRNA profiles from the OncoSG dataset were evaluated for expression of HGF, which was related to (A) the M1 macrophage marker CCR7 as well as the M2-associated cytokines (B) TGFB2 and (C) IL10. The Spearman’s Correlation Coefficient (D) and P value of all potential interactions are displayed above. (TIF) [file pone.0252197.s001.tif]
